# Estimating the incidence of unintended births and pregnancies at the sub-state level to inform program design

**DOI:** 10.1371/journal.pone.0240407

**Published:** 2020-10-15

**Authors:** Keith Kranker, Sarah Bardin, Dara Lee Luca, So O’Neil

**Affiliations:** Mathematica, Cambridge, Massachusetts, United States of America; University of Mississippi Medical Center, UNITED STATES

## Abstract

**Objectives:**

Unintended (mistimed or unwanted) pregnancies occur frequently in the United States and have negative effects. When designing prevention programs and intervention strategies for the provision of comprehensive birth control methods, it is necessary to identify (1) populations at high risk of unintended pregnancy, and (2) geographic areas with a concentration of need.

**Methods:**

To estimate the proportion and incidence of unintended births and pregnancies for regions in Missouri, two machine-learning prediction models were developed using data from the National Survey of Family Growth and the Missouri Pregnancy Risk Assessment Monitoring System. Each model was applied to Missouri birth certificate data from 2014 to 2016 to estimate the number of unintended births and pregnancies across regions in Missouri. Population sizes from the American Community Survey were incorporated to estimate the incidence of unintended births and pregnancies.

**Results:**

About 24,500 (34.0%) of the live births in Missouri each year were estimated to have resulted from unintended pregnancies: about 25 per 1,000 women (ages 15 to 45) annually. Further, 40,000 pregnancies (39.7%) were unintended each year: about 41 per 1,000 women annually. Unintended pregnancy was concentrated in Missouri’s largest urban areas, and annual incidence varied substantially across regions.

**Conclusions:**

Our proposed methodology was feasible to implement. Random forest modeling identified factors in the data that best predicted unintended birth and pregnancy and outperformed other approaches. Maternal age, marital status, health insurance status, parity, and month that prenatal care began predict unintended pregnancy among women with a recent live birth. Using this approach to estimate the rates of unintended births and pregnancies across regions within Missouri revealed substantial within-state variation in the proportion and incidence of unintended pregnancy. States and other agencies could use this study’s results or methods to better target interventions to reduce unintended pregnancy or address other public health needs.

## Background

The ability to freely decide and successfully plan the number, spacing, and timing of pregnancies is a fundamental human right, as first recognized by the 1968 International Convention on Human Rights and supported by multiple organizations thereafter, including the United Nations Sustainable Development Goals [1, 2]. However, more than 40 years later, 2.8 million women in the United States still experience an unintended (mistimed or unwanted) pregnancy, comprising 45% of all pregnancies in 2011 [3]. Research has shown that when an unintended pregnancy results in a birth, the infants tend to have worse outcomes than others. In particular, mothers experiencing an unintended pregnancy are less likely to seek early prenatal care and their babies are less likely to be breastfed and more likely to be of low birth weight [4]. Beyond the family, the costs to society of unintended pregnancy are substantial; one study estimated that costs of unintended pregnancy were at least $21 billion in 2010, or half of the costs spent on publicly funded pregnancies [5].

Rates of unintended pregnancies vary substantially across the United States with some areas, notably states in the New England region, exhibiting as few as 36 unintended pregnancies per 1,000 women ages 15 to 44, while areas in parts of the Southern and Western United States experience more than 60 unintended pregnancies per 1,000 women ages 15 to 44 [6]. Such variation is not surprising given the differences in sociodemographics, policies, and health care access across states. This variation does, however, suggest the need for state level programming tailored to the context of each state, which in turn requires further understanding of sub-state trends to support such decision-making around where, how, and to whom to roll out programming focused on reducing unintended pregnancy.

Although several data sources, such as the National Survey of Family Growth (NSFG) or Pregnancy Risk Assessment Monitoring System (PRAMS), can provide state or national estimates of unintended pregnancy [3, 7], there is currently no data that provides these estimates at a sub-state level—data which are necessary in order to design tailored state unintended pregnancy interventions, allocate resources, and make other policy decisions [3, 6, 8]. To our knowledge, there have not been any methods to enable decisionmakers to estimate in incidence of unintended births and pregnancies at sub-state levels using extant data sources. To support decision makers interested in developing interventions tailored to within state contexts, we developed a prediction modelling approach to assess sub-state levels of unintended pregnancy (that is, small area estimates) using the state of Missouri as a case study.

## Methods

### Study design

**Fig 1:**
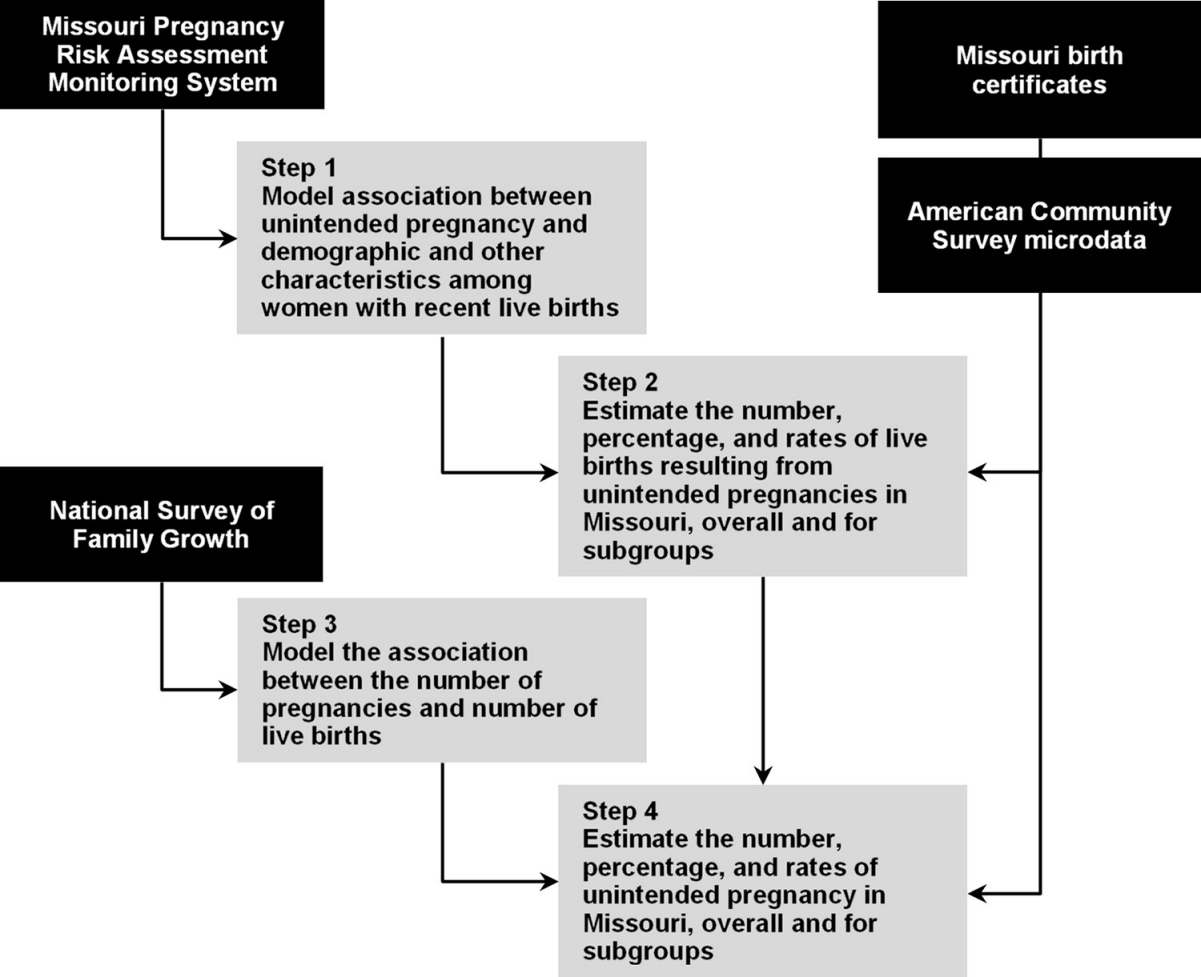
Data sources and analysis steps.

This study involved secondary analysis of extant survey and administrative data to develop and implement a feasible methodology for producing small area estimates of the proportion and incidence of unintended births and pregnancies. The analysis comprised four steps and drew data from four sources. Fig 1 summarizes the process and the sources used.

### Setting

We focus on women of childbearing age in the state of Missouri, from 2012 to 2016.

### Data

To estimate unintended pregnancy for regions within Missouri, we leveraged information from four data sources. All data files were the latest available at the time of analysis. The data sources were:

1. **Missouri PRAMS data for 2012 to 2015.** Using the state’s birth certificate data, PRAMS surveys a representative sample of women who recently delivered a live-born infant in the state. Forty-seven states currently collect data for PRAMS and report these data to the U.S. Centers for Disuse Control and Prevention (CDC). The survey asks sampled women about maternal behaviors, attitudes, and experiences before, during, and shortly after pregnancy. PRAMS was used because this is the standard data source for measuring rates of unintended pregnancy among live births at the state and national levels [3, 4, 9]. The sampling methodology and questionnaire have been documented by the CDC [10]. PRAMS is a mixed-mode surveillance system that relies on mail as the primary data collection mode, with telephone follow-up for mail non-respondents. Most states use health department staff to conduct mail survey operations; however, several states contract out the telephone portion to professional survey research organizations [11]. In total the 2012–2015 Missouri PRAMS collected data from 8,488 women. Among these respondents, 4,233 provided information regarding their pregnancy intention and were included in the analysis.
2. **Birth certificate data from Missouri for 2014 to 2016.** The birth certificate data, which included all live births in Missouri from 2014 to 2016, were obtained from the Missouri DHSS, Bureau of Vital Records. In total, there were 216,320 births during this timeframe. The data included items from the U.S. Standard Birth Certificate (2003 revision) [12] as well as the latitude and longitude coordinates for each record. The Bureau determined the geographic location for each birth record by geocoding the addresses provided on the birth certificate data. For 139 births the latitude and longitude were missing; these cases were excluded from the analysis, leaving a final sample size of 216,181 births.
3. **NSFG data for 2013 to 2015.** The NSFG is administered by the CDC, National Center for Health Statistics. The best publicly available data source on unintended pregnancy, the NSFG is administered to a nationally representative sample of women 15–44 years of age in the civilian, noninstitutionalized population of the United States [13]. Survey respondents give a history of all their pregnancies (if any), including the date of each pregnancy, and whether each pregnancy was unintended. Respondents provided a history of all their pregnancies (if any), including the date of each and whether each pregnancy was unintended and resulted in a live birth. In the 2013–2015 NSFG, 5,687 women reported data on 9,358 pregnancies. We focused on the respondents with at least one pregnancy and excluded 249 pregnancies where the woman was pregnant at the time of the interview, leaving a sample of 3,476 respondents and 9,109 pregnancies. The NSFG is the only nationally representative dataset of women of childbearing age in the United States that collects information on both unintended pregnancy and birth, which is why we chose to use it. NSFG is conducted through in-person interview, with a portion of the more sensitive questions answered privately by self-administration. The interviews are voluntary and confidential. The National Center for Health Statistics has contracted with the University of Michigan to conduct interviews for this study. Professional female interviewers from the University of Michigan’s Survey Research Center conduct in-home in-person interviews with eligible respondents [14].
4. **Public Use Microdata from the American Community Survey (ACS) 2016 5-year estimates.** The ACS public use microdata contains a sample of individual-level responses to the ACS between 2012 and 2016, representing approximately five percent of the U.S. population. The ACS is conducted on an ongoing basis and published annually and provides information related to employment, educational attainment, housing, and sociodemographics as well as geographic identifiers. The 2016 5-year estimates included a representative sample of 55,795 women ages 15 to 45 in Missouri, residing in 47 public-use microdata areas (PUMAs). A PUMA is a statistical unit of area that contains 100,000 or more residents. On average a PUMA in Missouri contained 21,000 women but this number varied from a low of 15,000 to a high of 36,000.

### Data analyses

#### Step 1. Modeling the associations between unintended pregnancy and demographics and other characteristics

To identify predictors of unintended pregnancy, we modeled the associations between unintended pregnancy and demographic and other characteristics in a sample of women who gave birth in Missouri using PRAMS data. We defined a pregnancy as unintended (*Y* = 1) if the woman responded, “I wanted to be pregnant later,” or “I didn’t want to be pregnant then or at any time in the future,” to the question “Thinking back to just before you got pregnant with your new baby, how did you feel about becoming pregnant?” We defined a pregnancy as not unintended (*Y* = 0) if the woman responded, “I wanted to be pregnant sooner,” “I wanted to be pregnant then,” or “I wasn’t sure what I wanted” (Table 1). The literature typically classifies the “unsure” response as “not unintended” [15, 16] and this practice was confirmed in recent research [17].

**Table 1:** Definition of the unintended pregnancy outcome measure.

Response to question: “Thinking back to just before you got pregnant with your new baby, how did you feel about becoming pregnant?”	Coded as
I wanted to be pregnant later	Unintended pregnancy
I wanted to be pregnant sooner	Not unintended pregnancy
I wanted to be pregnant then	Not unintended pregnancy
I didn’t want to be pregnant then or at any time in the future	Unintended pregnancy
I wasn’t sure what I wanted	Not unintended pregnancy

We aimed to develop a model that could predict intendedness (*Y*_*i*_) for a given live birth (*i*) as a function of observed covariates (*X*_*i*_): Pr(*Y*_*i*_ = 1|*X*_*i*_) = *f*(*X*_*i*_). We considered demographic and other characteristics that were available in both PRAMS and birth certificate data for inclusion in the model (Table 2). We explored various modeling techniques, including logistic regression, random forest, multilayer perceptron classifier neural network, gradient boosting, and linear classifier with stochastic gradient descent training [18], and chose the model with the highest area under the receiver curve, or *c-*statistic. We used K-fold cross-validation to assess the model’s out-of-sample predictive performance when comparing alternative models and choosing tuning parameters for the models. We applied the PRAMS survey weights to respondents’ data to produce state-representative results.

**Table 2:** Demographics and other characteristics included in the predictive models.

Characteristic	Used to estimate the probability that a live birth resulted from an unintended pregnancy ($\hat{f}()$ in Steps 1–2) ^a^^, ^^b^	Used to estimate the probability that a pregnancy resulted in a live birth ($\hat{g}()$ in Steps 3–4) ^b^^, ^^c^
Mother’s age	✔	✔
Mother’s race and ethnicity	✔	✔
Mother’s education (level completed)	✔	
Mother married	✔	✔
Mother was foreign born		✔
Insurance (private versus other or no insurance) ^d^	✔	
Pregnancy was unintended	n/a	✔
Pregnancy resulted from infertility treatment	✔	
Parity	✔	✔
Plurality (twins, triplets, etc.)	✔	
Prenatal care (adequacy of prenatal care utilization index, month of pregnancy in which prenatal care began, number of prenatal care visits)	✔	
Cigarette smoking (smoking before pregnancy or in first, second, or third trimester, smoking cessation)	✔	
Prepregnancy body mass index and recommended maternal weight gain	✔	
Pregnancy risk factors (prepregnancy diabetes, gestational diabetes, prepregnancy hypertension, gestational hypertension, eclampsia, or any of the above)	✔	
Previous adverse birth outcome (previous cesarean delivery, preterm birth, or poor pregnancy outcome)	✔	
Infant’s health at birth (birth weight, gestation <39 weeks, transferred within 24 hours of delivery, small or large for gestational age)	✔	
Breastfeeding at discharge	✔	
Infant’s gender	✔	
Father’s characteristics (education, race, ethnicity)	✔	

^a^ The model in Step 1 used variables available in both the Pregnancy Risk Assessment Monitoring System data and the Vital Records birth certificate data.

^b^ We imputed missing data for binary values as zeros and added a dummy indicator for the missing values. For continuous variables, we replaced the missing value with the median value of the variable, and added a corresponding missing indicator.

^c^ The model in Step 3 used variables available in both the National Survey of Family Growth data and the Vital Records birth certificate data.

^d^ Because of privacy concerns, Medicaid, other, and no insurance were combined into one category in the raw data files.

#### Step 2. Estimating the number and percentage of live births resulting from unintended pregnancies, by geographic region

We next identified Missouri geographic regions with the highest percentage of women with unintended births, defined as a live birth resulting from an unintended pregnancy also referred to as “unintended births” [9]. Broadly, this was done by applying the estimated model of unintended pregnancy from Step 1 $\left(\hat{f}()\right)$ to Missouri’s birth certificate data and then aggregating the results to estimate the number and percentage of births from unintended pregnancies in each region. We applied the model to estimate the probability that each live birth in Missouri (*b*) was the result of an unintended pregnancy, conditional on the characteristics of the mother and the family: ${\hat{Y}}_{b}=\hat{f}(X_{b})$.

We next aggregated the birth-specific probabilities of an unintended pregnancy by PUMA. We determined that PUMAs represented an appropriate level of aggregation for these analyses, as they were specific enough to provide action-oriented information but large enough (in population terms) for rates to be estimated with reasonable precision. There are 47 PUMAs in Missouri. In rural areas of the state, PUMAs consist of one or more counties, whereas counties in large urban areas contain multiple PUMAs. Women were assigned to PUMAs based on the geographic coordinates (latitude and longitude) of their residence, with addresses having been geocoded by Missouri’s Bureau of Vital Records.

We aggregated the birth-specific probabilities of an unintended pregnancy to estimate three outcomes for residents of each PUMA (overall and for specific age categories): (1) the average number of unintended births per year $\left(\frac{1}{3}\sum_{b}{\hat{Y}}_{b}\right)$; (2) the percentage of live births from unintended pregnancies $\left(\frac{1}{B}\sum_{b}{\hat{Y}}_{b}\right)$; and (3) the incidence of unintended births per year $\left(\frac{1}{3}\sum_{b}{\hat{Y}}_{b}/N\right)$, where we have 3 years of data, *B* = number of live births, and *N* = number of women who live in the PUMA according to ACS microdata. We present the final results in choropleth maps developed using ArcGIS 10.6, with darker-shaded areas identify regions with higher percentages or numbers.

We also applied a heat map (kernel density estimation) algorithm to the geocoded address data to construct a map with areas shaded according to the density of unintended pregnancies. In the heat map, darker-shaded areas identify regions with more unintended births per square mile per year. A kernel density algorithm was used to calculate the density of unintended pregnancies, using a search radius based on the mean area per unintended pregnancy. We used a search radius of 2.4 miles, as determined based on the mean area per each event. The mean area per each event is determined by taking the area of the state of Missouri and dividing by the number of unintended pregnancies/births. The distance is then calculated by taking the square root of 2 times this number, which research [19] suggests is an optimal distance to use for determining the density of points.

#### Step 3. Modeling the association between the number of pregnancies and the number of live births

As an interim step of estimating the number of unintended *pregnancies*, we modeled the ratio between the number of live births and the underlying number of pregnancies, assessing how this association varied based on women’s characteristics in a nationally representative sample of women of childbearing age in the United States. This step used the NSFG data described above. Our model weighted the data to account for the survey’s complex design.

For each pregnancy in the NSFG data (*j*), we developed a model of the probability that a pregnancy resulted in a live birth (*L*_*j*_ = 1) as a function of observed covariates (*Z*_*j*_) and intendedness (*Y*_*j*_): Pr (*L*_*j*_ = 1|*Z*_*j*_,*Y*_*j*_) = *g*(*Z*_*j*_,*Y*_*j*_). We considered several modeling techniques and used cross validation to choose the approach that best detected how the likelihood of a live birth varied (1) between intended and unintended pregnancies, and (2) by demographic and other characteristics. The covariates, *Z*_*j*_, are listed in Table 2. Because the Vital Records birth certificate and the NSFG share relatively few variables in common, this step contains fewer characteristics than were in the model in Step 1.

#### Step 4. Estimating the number and percentage of pregnancies that are unintended, by geographic region

To estimate the number and rates of unintended pregnancy in geographic regions in Missouri, we applied the estimated model of live births from Step 3 to Missouri’s birth certificate data. That is, our estimates are driven mainly by the estimated number of unintended births in the birth certificate file, with a multiplier applied to extrapolate the number of pregnancies. In this sample, about 71% of all pregnancies result in a live birth, giving a ratio of 1 live birth to 1.4 pregnancies, on average. This multiplier varies across subpopulations, based on (1) the woman’s particular demographic and other characteristics as reported on the birth certificate (*Z*_*b*_), and (2) the associations between unintended pregnancy and respondent characteristics observed in the NSFG data $\left(\hat{g}()\right)$. Specifically for each live birth (*b*) in the birth certificate data, we estimated the underlying number of unintended pregnancies as ${\hat{U}}_{b}=\frac{\hat{f}(X_{b})}{\hat{g}(Z_{b},1)}$ and the underlying total number of pregnancies as ${\hat{P}}_{b}=\frac{\hat{f}(X_{b})}{\hat{g}(Z_{b},1)}+\frac{1-\hat{f}(X_{b})}{\hat{g}(Z_{b},0)}$.

Finally, we aggregated these birth-specific quantities to estimate four outcomes for each PUMA (overall and by age group): (1) the average number of pregnancies per year $\left(\frac{1}{3}\sum_{b}{\hat{P}}_{b}\right)$, (2) the average number of unintended pregnancies per year $\left(\frac{1}{3}\sum_{b}{\hat{U}}_{b}\right)$, (3) the percentage of pregnancies that were unintended $(\sum_{b}{\hat{P}}_{b}/\sum_{b}{\hat{U}}_{b}),$ and (4) the incidence of unintended pregnancies per year $\left(\frac{1}{3}\sum_{b}{\hat{U}}_{b}/N\right)$. These data were shown in choropleth maps, where darker-shaded areas identify regions with higher percentages or numbers of births or pregnancies. We also constructed a heat map using the same approach as in Step 2, this time determining to use a search radius of 1.9 miles.

### Ethical consideration

Research protocols were approved by the Missouri Department of Health and Senior Services (DHSS) Internal Review Board (protocol #1357). Because this was a secondary data analysis of existing administrative data, informed consent was neither required nor obtained for the birth certificate data. Informed consent was obtained at the time data collection for the PRAMS and NSFG surveys. We complied with ethical practices in performing this study.

## Results

### Demographic and other factors associated with unintended pregnancy in Missouri

With a cross-validated *c-*statistic of 0.71, the random forest classifier algorithm outperformed other approaches at predicting unintended pregnancy in a sample of women who gave birth in Missouri in Step 1 of the methodology (S1 Table). In this sample, 34% of the women reported that their pregnancy was unintended.

**Fig 2:**
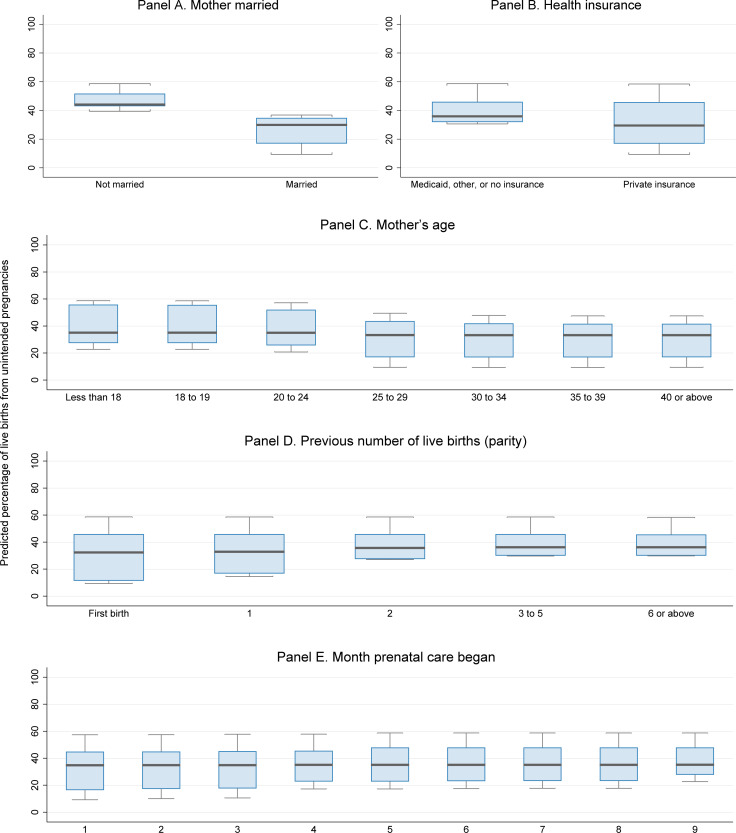
Estimated associations between mothers’ characteristics and the percentage of live births that resulted from unintended pregnancies.

Collectively, five variables account for 95% of the model’s ability to predict unintended pregnancy among women with a recent live birth, and the rest of the variables in the model contribute the remaining 5% (as measured by Gini importance). Fig 2 shows the magnitude of the association between unintended pregnancy and these five most important characteristics. Each panel simulates the rates of unintended pregnancy predicted by the random forest model if everyone in the birth certificate file had a specific characteristic. Specifically, the predictive margins were calculated by applying the model at fixed values of the characteristic of interest and averaging over the remaining covariates. The boxes indicate the 25th, 50th, and 75th percentiles, and the whiskers indicate the lower and upper adjacent values. These are known as partial dependence plots because they demonstrate the association between unintended pregnancy and a single characteristic of interest. This is important because variables associated with unintended pregnancy tend to be correlated.

Fig 2 demonstrates associations between with the percentage of live births that resulted from unintended pregnancies and the following five characteristics:

1. Among women with recent births, those who are not married are 10 percentage points more likely than those who are married to say the pregnancy was unintended, all else equal (Fig 2, Panel A). The model estimates the percentage of live births resulting from unintended pregnancies to be about 34% in Missouri’s birth certificate file, accounting for 59% of the women being unmarried in the data. However, if all the women had been unmarried (instead), then 47% of births would have been from unintended pregnancies (all else equal). In contrast, if all the women were married, the percentage would have been 37%, a 10-percentage-point difference.
2. Having private health insurance is associated with an 8-percentage-point lower likelihood of a live birth being from an unintended pregnancy, compared to having Medicaid, other, or no insurance (Panel B). This may be because mothers with private health insurance have higher socioeconomic status, better access to contraceptive care and abortion services, or other advantages.
3. Other factors held constant, unintended pregnancies are more common among young mothers than among somewhat older mothers. The estimated percentage of live births resulting from unintended pregnancies is more than 40% for mothers under age 20, but 30 to 32% for women aged 25 and older (Panel C).
4. After adjusting for other factors, having two or more previous births is associated with a 6- to 8-percentage-point higher chance of a live birth being from an unintended pregnancy, relative to mothers who had no more than one previous birth (Panel D).
5. All else equal, the estimated percentage of live births resulting from unintended pregnancies is 5 to 7 points lower for mothers who began prenatal care in the first few months of their pregnancies.

In robustness analyses, we reestimated this model using the additional PRAMS data from Missouri’s bordering states. The resulting model had slightly worse predictive power for births in Missouri, so we do not report results with the PRAMS data from these additional states.

### Unintended births in Missouri

The birth certificate data include 216,181 live births in Missouri from 2014 to 2016, or 72,000 a year. Our model predicted that about 24,500 (34.0%) resulted from unintended pregnancies. This indicates an estimated annual incidence of about 25 unintended births per 1,000 women (aged 15 to 45) per year in Missouri.

**Fig 3:**
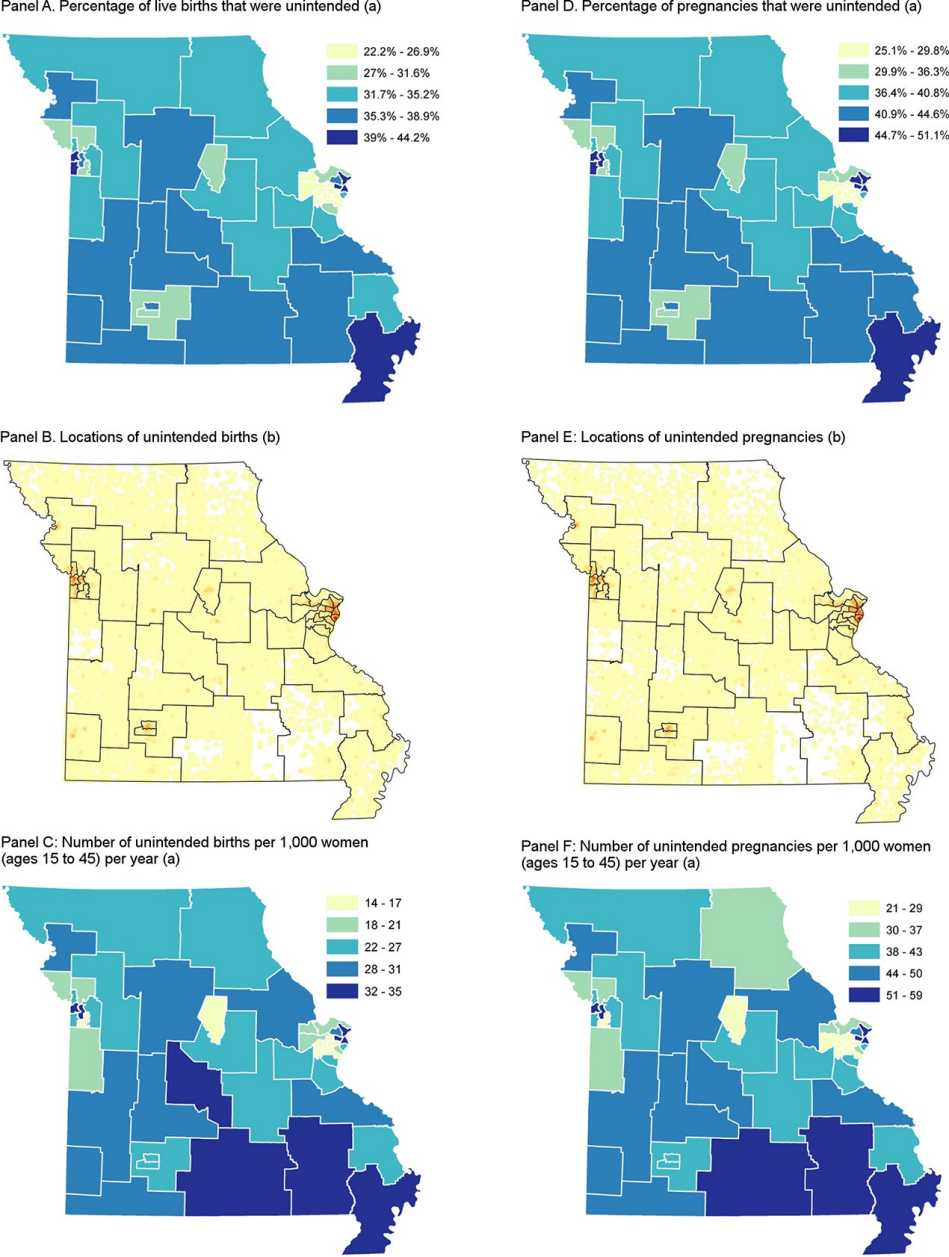
Unintended births and pregnancies (estimated) in Missouri, by region, 2014 to 2016.

The characteristics of women varied across regions in Missouri, as did population size and overall fertility rates. Consequently, the predicted numbers and incidence of unintended births varied substantially across regions (Fig 3, Panels A–C). In some areas, less than 25% of live births were unintended, but in six areas, more than 40% were (Panel A). The density of unintended births was highest in urban areas such as Kansas City, St. Louis, and Springfield (Panel B). Annual incidence also varied across regions, from 14 to 35 unintended births per 1,000 women (aged 15 to 45) (Panel C). The annual incidence varied substantially across age groups, with the highest rates among women ages 18 to 30 (S2 and S3 Tables).

### Unintended pregnancies in Missouri

The random forest classifier outperformed other approaches in terms of the accuracy of the prediction (cross-validated *c-*statistic = 0.66), and revealed three variables that predicted most strongly whether a pregnancy resulted in a live birth: (1) whether the pregnancy was unintended, (2) whether the mother was married, and (3) the mother’s age.

Applying the model to the birth certificate file suggests that Missouri had roughly 100,700 pregnancies per year, about 40,000 (or 39.7%) of which were unintended. The percentage of pregnancies that are unintended is higher than the percentage of live births resulting from unintended pregnancies (34%) because unintended pregnancies are less likely to result in a live birth than other pregnancies.

Our results suggest that unintended pregnancy was not distributed uniformly across regions (Fig 3, Panels D–F). Kansas City, St. Louis, and the southeast corner of Missouri had the highest estimated percentage of pregnancies that were unintended (Panel D). In several areas near St. Louis, more than half of pregnancies were estimated to be unintended, double the rate in other areas. Unintended pregnancies were predicted to be concentrated in urban areas (Panel E). Many of the areas with higher incidence of unintended pregnancy had higher incidence of unintended births. There were seven areas with at least 50 unintended pregnancies per 1,000 women per year.

These numbers translate into an annual rate of about 41 unintended pregnancies per 1,000 women (aged 15 to 45). The annual incidence of unintended pregnancy varied across regions (Panel F), and nine regions had more than 50 unintended pregnancies per 1,000 women (aged 15 to 45). The incidence of unintended pregnancy was highest for women aged 21 to 29.

## Discussion

About 24,500 (34.0%) of the live births in Missouri each year were estimated to have resulted from unintended pregnancies: about 25 per 1,000 women (aged 15 to 45) annually. Further, 40,000 pregnancies (39.7%) were unintended each year: about 41 per 1,000 women annually. Unintended pregnancy was concentrated in Missouri’s largest urban areas, and annual incidence varied substantially across regions.

Our estimate of 41 unintended pregnancies per 1,000 women in Missouri, based largely on 2012–2015 PRAMS data, is lower than a widely cited estimate of 46 unintended pregnancies per 1,000 women based on 2010 PRAMS data for Missouri [4]. The difference is most likely explained by a combination of three factors. First, this report uses different data sources and methods to extrapolate from the number of births to the number of pregnancies. Specifically, this report uses information from the NSFG to build a machine-learning algorithm to estimate the number of unintended pregnancies represented by an unintended birth, whereas the other estimate was calculated using a national estimate on the number of abortions and fetal losses that resulted from an unintended pregnancy to estimate unintended pregnancies within the state of Missouri. Second, the PRAMS survey questionnaire was updated beginning in 2012 to include an additional response category to the pregnancy intentionality question (“I wasn’t sure what I wanted”), which made it impossible to directly compare data collected from the older and newer survey instruments. When this response option is coded as “not unintended” the percentage of PRAMS respondents with unintended pregnancies is 34%. If this response option had instead been coded as “unintended” the rate would have been 52%, and the rate would have been 41% if respondents with this response option had been dropped from the analyses (data not shown). Third, the percentage of births resulting from unintended pregnancies has been decreasing, dropping by 7 percentage points from 2012 to 2015 in Missouri, according to the PRAMS data we used. Considering the differences in year of estimate, our study estimates falls within the plausible range. Furthermore, another study by Finer and Zolna (2016) [3] using NSFG data estimated the rate of unintended pregnancy in the United States at 45 per 1,000 women with a decline of 18% between 2008 and 2011. A similar 18% decline, applied to the Missouri-specific estimate of 46 unintended pregnancies per 1,000 women in 2010, would result in an approximate rate of 39 unintended pregnancies per 1,000 women in 2013—close to our estimate of 41.

We chose to use random forest because it performed the best among various modeling techniques. Specifically, random forest produced a higher cross-validated area under the receiver curve, or *c*-statistic, than logistic regression, multilayer perceptron classifier neural network, gradient boosting, and linear classifier with stochastic gradient descent training. The improved performance of random forest models over more traditional parametric modeling methods (logistic regression in this case) is not altogether surprising. Random forest and other machine learning models are increasingly utilized in public health studies (for example, Mooney and Pejaver [20] provide an in-depth discussion.) Researchers may wish to consider random forest modeling when working with PRAMS and NSFG data in future work.

The associations we found which predict unintended births overlap with, but are different from, other studies [4, 8, 15, 21]. Our study identified five key predictive factors of unintended pregnancy in the United States: (1) marital status, (2) health insurance, (3) age, (4) parity, and (5) month prenatal care began. Two factors associated with unintended pregnancy—lacking private insurance and receiving prenatal care later—are not as commonly discussed in the literature. Further, race, ethnicity, and education—which have previously been identified in the literature as key factors—are not among the key predictors in our model. That is, they played a relatively minor role in predicting unintended births after accounting for the associations between unintended pregnancy and the five key predictors. These findings highlight that data-driven methods can identify key predictors of unintended pregnancy that, a priori, may be less obvious. Specifically, random forest modeling helps identify the individual factors, and combinations of factors, that best predicted unintended birth and pregnancy in a particular state context.

As demographics and other characteristics of women with live births and fertility rates vary across a state, our model translates that variation into more accurate estimates of unintended pregnancy at the sub-state level. Specifically, our study finds substantial variation in the proportion and incidence of unintended births and pregnancies across regions. Our findings are subject to at least four limitations. First, there is the potential for recall and nonresponse bias in the PRAMS and NSFG data. In particular, studies have shown that information on abortion (both induced and spontaneous) is likely underreported in the NSFG due to social stigma [22–24]. As a result, our estimates of unintended pregnancies are likely underestimated. Differential underreporting for women with different characteristics is of particular concern. Second, because the number and rates of live births, as well as the characteristics of women, vary across geographic regions, so do the estimated rates of unintended births and pregnancies generated by our approach; however, the associations between outcomes and characteristics of women were held constant across the entire state—even though those associations could have varied across regions. One model was based on a nationwide sample, with a caveat that associations for Missouri might differ from those for the nation as a whole. That is, we had to use nationally representative data since no other data sources had a large enough sample to permit assessment of these associations within Missouri. Third, the estimates do not include Missouri residents who gave birth out of state or nonresidents who gave birth in Missouri. Fourth, small sample sizes prevented the analysis of unintended pregnancy for smaller geographic units (for example, census tract or block group). Finally, we could not estimate confidence intervals for our estimates, due in part to a lack of consensus on how to compute confidence intervals for machine learning models and computational limitations.

## Conclusion

Empowering women to choose whether and when to have children can have many benefits to everyone, including women, children, and families’ health and well-being, and to society in terms of reducing health care and associated costs [25–27]. Unfortunately, unintended pregnancies remain common in the United States [3]. As a result, various government agencies, philanthropic institutions, and community-based organizations have launched initiatives to reduce unintended pregnancy in many states, including Delaware, Colorado, Iowa, Missouri, South Carolina, and Utah. These entities would benefit from having data to understand rates of unintended pregnancy at community levels to inform these initiatives design, rollout, ongoing implementation, and assess potential effect within the state. Until now, estimates were currently available only at state and national level, however. Ultimately, our study represents a novel approach to obtaining small area estimates for community-level insights. Sophisticated models have been used to combine survey and geocoded administrative data to obtain estimates for small geographic areas in other contexts (for example, [28, 29]), but this appears to be the first time they have been used to obtain insights on within-state populations and regions burdened by unintended pregnancy. Although we focus on unintended pregnancy in Missouri, these methods could potentially be adapted to other states using national data from NSFG and state-level PRAMS and birth certificate data, or adapted to other public health conditions where sub-state level data are not readily available.

As applied to unintended pregnancy, states and other agencies could use the results or methods from this study, especially in conjunction with other data sources, to support their efforts to better target interventions to reduce unintended. For example, if a state seeks to increase the availability of long-acting reversible contraception, the policy or program implementers might consider targeting health care providers in regions that have high rates of unintended pregnancy along with low provision of long-acting contraception. Thus, it may be helpful to combine these results with other data sources which identify the characteristics and capabilities of health care providers in Missouri.

## Supporting information

S1 Table Cross-validated model performance of selected modeling approaches. (DOCX)

S2 Table Estimated numbers of unintended births and pregnancies in Missouri, by region, 2014 to 2016. (DOCX)

S3 Table Estimated incidence of unintended births and pregnancies among women in Missouri aged 15 to 45, by region, 2014 to 2016. (DOCX)
