# Supplementary material for: Estimating the incidence of unintended births and pregnancies at the sub-state level to inform program design
Source: PLoS One. 2020 Oct 15;15(10):e0240407. doi: 10.1371/journal.pone.0240407 (PMC7561158; doi:10.1371/journal.pone.0240407)
Supplement: S1 Table — (DOCX) [file pone.0240407.s001.docx]

**S1 Table. Cross-validated model performance of selected modeling approaches.**

| **Classifier (model type) and key turning parameters ^a^** | ***c-*statistic** |
| --- | --- |
| **Model used to estimate the probability that a live birth  resulted from an unintended pregnancy (**$\hat{\boldsymbol{f}}\boldsymbol{( )}$ **in Steps 1-2)** | |
| **Random forest** |  |
| - min_samples_split=750, max_features=None | **0.7142** |
| - min_samples_split=10, max_features=None | 0.5969 |
| - min_samples_split=50, max_features=None | 0.6391 |
| - min_samples_split=100, max_features=None | 0.6598 |
| - min_samples_split=200, max_features=None | 0.6799 |
| - min_samples_split=300, max_features=None | 0.6927 |
| - min_samples_split=400, max_features=None | 0.7059 |
| - min_samples_split=500, max_features=None | 0.7098 |
| - min_samples_split=750, max_features=0.25 | 0.7065 |
| - min_samples_split=750, max_features=0.4 | 0.7092 |
| - min_samples_split=750, max_features=0.5 | 0.7104 |
| - min_samples_split=750, max_features=0.6 | 0.7104 |
| - min_samples_split=750, max_features=0.75 | 0.7117 |
| - min_samples_split=750, max_features=auto | 0.7033 |
| - min_samples_split=1000, max_features=None | 0.7078 |
| **Gradient boosting** |  |
| - min_samples_split=100, max_features=None, n_estimators=10 | 0.6985 |
| - min_samples_split=100, max_features=None, n_estimators=50 | 0.6106 |
| - min_samples_split=500, max_features=None, n_estimators=10 | 0.7070 |
| **Linear classifier with stochastic gradient descent** |  |
| - learning_rate=constant, loss=hinge, penalty=None | 0.6319 |
| - learning_rate=constant, loss=perceptron, penalty=None | 0.6153 |
| - learning_rate=constant, loss=perceptron, penalty=l2 | 0.5786 |
| - learning_rate=optimal, loss=hinge, penalty=None | 0.6417 |
| - learning_rate=optimal, loss=perceptron, penalty=None | 0.6149 |
| **Multilayer perceptron classifier neural network** |  |
| - activation=relu, hidden_layer_sizes=(50, 25, 10, 10, 10), solver=adam | 0.5889 |
| **Logistic regression** |  |
| **Model used to estimate the probability that a pregnancy  resulted in a live birth (**$\hat{\boldsymbol{g}}\boldsymbol{( )}$ **in Steps 3-4)** | |
| **Random forest** |  |
| - min_samples_split=500, max_features=0.5, min_samples_leaf=5 | **0.6553** |
| - min_samples_split=5, max_features=0.5, min_samples_leaf=5 | 0.6328 |
| - min_samples_split=10, max_features=0.5, min_samples_leaf=5 | 0.6329 |
| - min_samples_split=25, max_features=0.5, min_samples_leaf=5 | 0.6391 |
| - min_samples_split=50, max_features=0.5, min_samples_leaf=5 | 0.6458 |
| - min_samples_split=100, max_features=0.5, min_samples_leaf=5 | 0.6518 |
| - min_samples_split=150, max_features=0.5, min_samples_leaf=5 | 0.6538 |
| - min_samples_split=200, max_features=0.5, min_samples_leaf=5 | 0.6543 |
| - min_samples_split=250, max_features=0.5, min_samples_leaf=5 | 0.6548 |
| - min_samples_split=500, max_features=0.25, min_samples_leaf=5 | 0.6525 |
| - min_samples_split=500, max_features=0.4, min_samples_leaf=5 | 0.6551 |
| - min_samples_split=500, max_features=0.6, min_samples_leaf=5 | 0.6552 |
| - min_samples_split=500, max_features=0.75, min_samples_leaf=5 | 0.6548 |
| - min_samples_split=500, max_features=auto, min_samples_leaf=5 | 0.6526 |
| - min_samples_split=750, max_features=0.5, min_samples_leaf=5 | 0.6535 |
| - min_samples_split=500, max_features=0.5, min_samples_leaf=1 | 0.6551 |
| **Gradient boosting** |  |
| - min_samples_split=200, max_features=0.5 | 0.6378 |
| **Linear classifier with stochastic gradient descent** |  |
| - learning_rate=constant, loss=hinge | 0.6283 |
| - learning_rate=constant, loss=hinge, penalty=l2 | 0.6153 |
| - learning_rate=constant, loss=perceptron | 0.6249 |
| - learning_rate=constant, loss=perceptron, penalty=l2 | 0.6148 |
| - learning_rate=optimal, loss=hinge | 0.6268 |
| - learning_rate=optimal, loss=hinge, penalty=l2 | 0.6312 |
| - learning_rate=optimal, loss=perceptron | 0.6198 |
| - learning_rate=optimal, loss=perceptron, penalty=l2 | 0.6252 |
| **Multilayer perceptron classifier neural network** |  |
| - activation=relu, hidden_layer_sizes=(50, 25, 10, 10, 10), solver=adam | 0.5524 |
| **Logistic regression** | 0.6449 |

^a^ Tuning parameters refer to settings for the scikit-learn package for Python. We used 6-fold cross validation to test model performance, calculating the mean *c-*statistic (area under the receiver curve). We estimated more models than are presented in this table, but for brevity showed only a subset of the models.
