# Supplementary material for: Estimating the incidence of unintended births and pregnancies at the sub-state level to inform program design
Source: PLoS One. 2020 Oct 15;15(10):e0240407. doi: 10.1371/journal.pone.0240407 (PMC7561158; doi:10.1371/journal.pone.0240407)
Supplement: S2 Table — (DOCX) [file pone.0240407.s002.docx]

**S2 Table. Estimated numbers of unintended births and pregnancies in Missouri, by region, 2014 to 2016.**

| **Public use microdata area (PUMA)** | | **Live births** | | | | **Pregnancies** | | | |
| --- | --- | --- | --- | --- | --- | --- | --- | --- | --- |
|  |  | **Number of live births per year** | | | **Percentage of live births that were from unintended pregnancies** | **Number of pregnancies per year** | | | **Percentage of pregnancies that were unintended** |
|  |  | **Total number** | **Number resulting from unintended pregnancies** | **Number resulting from intended pregnancies** |  | **Total number** | **Number of unintended pregnancies** | **Number of intended pregnancies** |  |
| **State of Missouri** | | **72,093** | **24,524** | **47,569** | **34.0%** | **100,729** | **39,975** | **60,754** | **39.7%** |
| 00100 | Northwest Missouri | 1,272 | 444 | 828 | 34.9% | 1,770 | 714 | 1,055 | 40.4% |
| 00200 | Buchanan, Andrew & DeKalb Counties | 1,420 | 518 | 902 | 36.5% | 2,014 | 863 | 1,152 | 42.8% |
| 00300 | Northeast Missouri | 1,294 | 447 | 848 | 34.5% | 1,801 | 719 | 1,083 | 39.9% |
| 00400 | Lincoln, Warren, Audrain, Pike & Montgomery Counties | 1,827 | 636 | 1,191 | 34.8% | 2,574 | 1,051 | 1,523 | 40.8% |
| 00500 | Cole, Callaway, Moniteau & Osage Counties | 1,771 | 584 | 1,187 | 33.0% | 2,467 | 953 | 1,513 | 38.6% |
| 00600 | Boone County | 2,102 | 619 | 1,483 | 29.5% | 2,881 | 995 | 1,886 | 34.5% |
| 00700 | Pettis, Randolph, Saline, Cooper, Howard, Carroll & Chariton Counties | 1,622 | 578 | 1,044 | 35.6% | 2,266 | 936 | 1,330 | 41.3% |
| 00800 | Johnson, Lafayette, Ray, Clinton & Caldwell Counties | 1,650 | 549 | 1,101 | 33.3% | 2,282 | 884 | 1,398 | 38.7% |
| 00901 | Clay County (Northeast) | 1,215 | 345 | 870 | 28.4% | 1,661 | 551 | 1,110 | 33.2% |
| 00902 | Kansas City (North Central), Gladstone City & North Kansas City | 1,422 | 455 | 966 | 32.0% | 1,971 | 742 | 1,230 | 37.6% |
| 00903 | Platte County | 1,172 | 356 | 815 | 30.4% | 1,617 | 578 | 1,039 | 35.7% |
| 01001 | Jackson County--Kansas City (Central) | 2,316 | 983 | 1,332 | 42.5% | 3,302 | 1,606 | 1,696 | 48.6% |
| 01002 | Jackson County (North Central) | 1,579 | 614 | 965 | 38.9% | 2,256 | 1,022 | 1,234 | 45.3% |
| 01003 | Jackson County (East) | 1,569 | 513 | 1,056 | 32.7% | 2,186 | 839 | 1,346 | 38.4% |
| 01004 | Jackson County (South Central) | 1,041 | 288 | 754 | 27.6% | 1,413 | 454 | 959 | 32.1% |
| 01005 | Jackson County--Kansas City (South) | 1,669 | 669 | 1,001 | 40.0% | 2,385 | 1,104 | 1,282 | 46.3% |
| 01100 | Cass & Bates Counties | 1,008 | 350 | 658 | 34.7% | 1,413 | 572 | 841 | 40.5% |
| 01200 | Lawrence, Henry, Vernon, Cedar, Barton, St. Clair & Dade Counties | 1,430 | 519 | 911 | 36.3% | 2,000 | 838 | 1,161 | 41.9% |
| 01300 | Laclede, Polk, Benton, Dallas & Hickory Counties | 1,317 | 471 | 845 | 35.8% | 1,837 | 759 | 1,078 | 41.3% |
| 01400 | Pulaski, Camden, Miller & Morgan Counties | 1,783 | 644 | 1,139 | 36.1% | 2,461 | 1,017 | 1,444 | 41.3% |
| 01500 | Phelps, Crawford, Dent, Gasconade & Maries Counties | 1,212 | 420 | 792 | 34.7% | 1,693 | 685 | 1,009 | 40.4% |
| 01600 | Franklin County | 1,233 | 406 | 826 | 33.0% | 1,729 | 673 | 1,057 | 38.9% |
| 01701 | St. Charles County (South) | 1,281 | 324 | 956 | 25.3% | 1,733 | 512 | 1,221 | 29.5% |
| 01702 | St. Charles County (Northwest) | 1,702 | 457 | 1,245 | 26.9% | 2,317 | 729 | 1,588 | 31.4% |
| 01703 | St. Charles County (Northeast) | 1,530 | 429 | 1,101 | 28.0% | 2,102 | 695 | 1,407 | 33.0% |
| 01801 | St. Louis County (Northeast) | 1,665 | 736 | 929 | 44.2% | 2,457 | 1,255 | 1,202 | 51.1% |
| 01802 | St. Louis County (Northwest) | 1,647 | 637 | 1,010 | 38.7% | 2,372 | 1,072 | 1,299 | 45.2% |
| 01803 | St. Louis County (Inner Ring North) | 1,621 | 654 | 967 | 40.4% | 2,341 | 1,101 | 1,240 | 47.0% |
| 01804 | St. Louis County (West) | 1,081 | 266 | 815 | 24.6% | 1,449 | 412 | 1,038 | 28.4% |
| 01805 | St. Louis County (Central) | 1,202 | 266 | 935 | 22.2% | 1,608 | 403 | 1,205 | 25.1% |
| 01806 | St. Louis County (Central West) | 1,221 | 284 | 937 | 23.2% | 1,628 | 432 | 1,195 | 26.5% |
| 01807 | St. Louis County (Inner Ring South) | 1,965 | 502 | 1,463 | 25.5% | 2,670 | 797 | 1,874 | 29.8% |
| 01808 | St. Louis County (South) | 1,220 | 303 | 917 | 24.8% | 1,652 | 477 | 1,175 | 28.9% |
| 01901 | St. Louis City (North) | 1,781 | 775 | 1,006 | 43.5% | 2,611 | 1,312 | 1,300 | 50.2% |
| 01902 | St. Louis City (South) | 2,734 | 920 | 1,814 | 33.6% | 3,846 | 1,518 | 2,327 | 39.5% |
| 02001 | Jefferson County (North) | 1,333 | 414 | 919 | 31.0% | 1,866 | 688 | 1,178 | 36.9% |
| 02002 | Jefferson County (South) | 1,240 | 414 | 826 | 33.4% | 1,754 | 696 | 1,059 | 39.7% |
| 02100 | St. Francois, Washington, Perry & Ste. Genevieve Counties | 1,408 | 513 | 895 | 36.5% | 2,000 | 854 | 1,146 | 42.7% |
| 02200 | Cape Girardeau, Scott & Bollinger Counties | 1,502 | 529 | 973 | 35.2% | 2,113 | 870 | 1,243 | 41.2% |
| 02300 | Dunklin, Stoddard, New Madrid, Pemiscot & Mississippi Counties | 1,322 | 544 | 778 | 41.1% | 1,899 | 902 | 997 | 47.5% |
| 02400 | Butler, Ripley, Wayne, Madison, Iron, Reynolds & Carter Counties | 1,234 | 474 | 759 | 38.4% | 1,753 | 782 | 972 | 44.6% |
| 02500 | Howell, Texas, Wright, Douglas, Oregon, Ozark & Shannon Counties | 1,442 | 541 | 901 | 37.5% | 2,019 | 869 | 1,150 | 43.0% |
| 02601 | Christian, Greene (Outside Springfield City) & Webster Counties | 2,018 | 637 | 1,381 | 31.6% | 2,756 | 999 | 1,757 | 36.3% |
| 02602 | Greene County--Springfield City (North) | 1,612 | 599 | 1,013 | 37.2% | 2,288 | 992 | 1,296 | 43.4% |
| 02603 | Greene County--Springfield City (South) | 1,542 | 477 | 1,065 | 30.9% | 2,127 | 771 | 1,355 | 36.3% |
| 02700 | Taney, Barry, Stone & McDonald Counties | 1,534 | 579 | 955 | 37.7% | 2,148 | 934 | 1,214 | 43.5% |
| 02800 | Jasper & Newton Counties | 2,332 | 840 | 1,492 | 36.0% | 3,241 | 1,351 | 1,890 | 41.7% |
