# Supplementary material for: Estimating the incidence of unintended births and pregnancies at the sub-state level to inform program design
Source: PLoS One. 2020 Oct 15;15(10):e0240407. doi: 10.1371/journal.pone.0240407 (PMC7561158; doi:10.1371/journal.pone.0240407)
Supplement: S3 Table — (DOCX) [file pone.0240407.s003.docx]

**S3 Table. Estimated incidence of unintended births and pregnancies among women in Missouri aged 15 to 45, by region, 2014 to 2016.**

| **Public use microdata area (PUMA)** | | **Number of women** | **Number of live births from unintended  pregnancies per 1,000 women per year** | | | | | | **Number of unintended pregnancies  per 1,000 women per year** | | | | | |
| --- | --- | --- | --- | --- | --- | --- | --- | --- | --- | --- | --- | --- | --- | --- |
|  |  |  | **All women** | **Age group** | | | | | **All women** | **Age group** | | | | |
|  |  |  |  | **15−18** | **18−20** | **21−23** | **24−29** | **30−45** |  | **15−18** | **18−20** | **21−23** | **24−29** | **30−45** |
| **State of Missouri** | | **966,116** | **25** | **7** | **33** | **47** | **44** | **15** | **41** | **12** | **57** | **78** | **72** | **24** |
| 00100 | Northwest Missouri | 16,834 | 26 | 8 | 25 | 52 | 47 | 15 | 42 | 13 | 44 | 84 | 75 | 23 |
| 00200 | Buchanan, Andrew & DeKalb Counties | 18,266 | 28 | 9 | 39 | 58 | 54 | 13 | 47 | 16 | 68 | 97 | 91 | 21 |
| 00300 | Northeast Missouri | 19,620 | 23 | 6 | 17 | 39 | 47 | 14 | 37 | 9 | 30 | 64 | 75 | 22 |
| 00400 | Lincoln, Warren, Audrain, Pike & Montgomery Counties | 21,363 | 30 | 7 | 53 | 64 | 55 | 15 | 49 | 11 | 92 | 107 | 91 | 24 |
| 00500 | Cole, Callaway, Moniteau & Osage Counties | 22,637 | 26 | 6 | 25 | 57 | 53 | 15 | 42 | 11 | 44 | 95 | 87 | 23 |
| 00600 | Boone County | 36,422 | 17 | 5 | 9 | 15 | 29 | 17 | 27 | 9 | 16 | 25 | 47 | 26 |
| 00700 | Pettis, Randolph, Saline, Cooper, Howard, Carroll & Chariton Counties | 19,754 | 29 | 11 | 37 | 65 | 55 | 14 | 47 | 18 | 64 | 108 | 88 | 22 |
| 00800 | Johnson, Lafayette, Ray, Clinton & Caldwell Counties | 22,106 | 25 | 6 | 25 | 38 | 57 | 14 | 40 | 10 | 43 | 61 | 90 | 21 |
| 00901 | Clay County (Northeast) | 18,272 | 19 | 4 | 20 | 42 | 38 | 13 | 30 | 7 | 36 | 71 | 60 | 20 |
| 00902 | Kansas City (North Central), Gladstone City & North Kansas City | 18,120 | 25 | 7 | 34 | 43 | 38 | 17 | 41 | 11 | 59 | 73 | 63 | 26 |
| 00903 | Platte County | 17,020 | 21 | 3 | 26 | 42 | 38 | 15 | 34 | 5 | 46 | 70 | 62 | 23 |
| 01001 | Jackson County--Kansas City (Central) | 31,042 | 32 | 20 | 49 | 62 | 40 | 18 | 52 | 32 | 82 | 101 | 66 | 29 |
| 01002 | Jackson County (North Central) | 18,437 | 33 | 16 | 61 | 65 | 54 | 16 | 55 | 26 | 104 | 108 | 90 | 26 |
| 01003 | Jackson County (East) | 21,946 | 23 | 4 | 44 | 62 | 44 | 12 | 38 | 7 | 76 | 103 | 72 | 19 |
| 01004 | Jackson County (South Central) | 16,919 | 17 | 3 | 23 | 36 | 31 | 13 | 27 | 6 | 40 | 60 | 49 | 19 |
| 01005 | Jackson County--Kansas City (South) | 26,086 | 26 | 10 | 39 | 47 | 41 | 14 | 42 | 16 | 67 | 77 | 69 | 22 |
| 01100 | Cass & Bates Counties | 18,149 | 19 | 5 | 31 | 48 | 38 | 10 | 32 | 8 | 53 | 80 | 62 | 15 |
| 01200 | Lawrence, Henry, Vernon, Cedar, Barton, St. Clair & Dade Counties | 16,902 | 31 | 8 | 46 | 86 | 63 | 14 | 50 | 14 | 79 | 139 | 102 | 21 |
| 01300 | Laclede, Polk, Benton, Dallas & Hickory Counties | 15,442 | 31 | 10 | 42 | 57 | 68 | 14 | 49 | 17 | 72 | 91 | 108 | 22 |
| 01400 | Pulaski, Camden, Miller & Morgan Counties | 20,468 | 31 | 10 | 38 | 55 | 61 | 17 | 50 | 16 | 64 | 87 | 95 | 26 |
| 01500 | Phelps, Crawford, Dent, Gasconade & Maries Counties | 15,797 | 27 | 7 | 37 | 53 | 51 | 14 | 43 | 12 | 65 | 87 | 83 | 21 |
| 01600 | Franklin County | 15,925 | 26 | 6 | 48 | 54 | 39 | 14 | 42 | 10 | 85 | 92 | 65 | 22 |
| 01701 | St. Charles County (South) | 17,936 | 18 | 2 | 15 | 32 | 31 | 17 | 29 | 3 | 26 | 55 | 49 | 25 |
| 01702 | St. Charles County (Northwest) | 21,995 | 21 | 3 | 19 | 35 | 41 | 16 | 33 | 5 | 34 | 60 | 66 | 25 |
| 01703 | St. Charles County (Northeast) | 22,009 | 19 | 3 | 16 | 20 | 33 | 17 | 32 | 6 | 29 | 34 | 54 | 26 |
| 01801 | St. Louis County (Northeast) | 21,842 | 34 | 9 | 51 | 69 | 59 | 16 | 57 | 15 | 87 | 116 | 101 | 28 |
| 01802 | St. Louis County (Northwest) | 21,405 | 30 | 7 | 45 | 52 | 56 | 17 | 50 | 11 | 77 | 88 | 94 | 28 |
| 01803 | St. Louis County (Inner Ring North) | 18,518 | 35 | 11 | 51 | 81 | 51 | 20 | 59 | 18 | 87 | 136 | 86 | 33 |
| 01804 | St. Louis County (West) | 16,800 | 16 | 1 | 13 | 16 | 23 | 17 | 25 | 2 | 22 | 27 | 36 | 25 |
| 01805 | St. Louis County (Central) | 18,989 | 14 | 1 | 3 | 8 | 17 | 19 | 21 | 1 | 6 | 13 | 27 | 28 |
| 01806 | St. Louis County (Central West) | 17,423 | 16 | 1 | 8 | 15 | 27 | 18 | 25 | 1 | 13 | 25 | 42 | 26 |
| 01807 | St. Louis County (Inner Ring South) | 23,901 | 21 | 2 | 17 | 34 | 30 | 20 | 33 | 4 | 30 | 59 | 49 | 30 |
| 01808 | St. Louis County (South) | 19,056 | 16 | 1 | 13 | 14 | 25 | 17 | 25 | 2 | 24 | 25 | 41 | 25 |
| 01901 | St. Louis City (North) | 24,829 | 31 | 12 | 38 | 56 | 44 | 17 | 53 | 20 | 64 | 92 | 75 | 29 |
| 01902 | St. Louis City (South) | 36,318 | 25 | 11 | 44 | 54 | 31 | 17 | 42 | 18 | 74 | 90 | 51 | 28 |
| 02001 | Jefferson County (North) | 18,021 | 23 | 6 | 35 | 43 | 46 | 13 | 38 | 11 | 63 | 74 | 78 | 21 |
| 02002 | Jefferson County (South) | 16,892 | 24 | 6 | 34 | 49 | 50 | 13 | 41 | 10 | 61 | 84 | 84 | 21 |
| 02100 | St. Francois, Washington, Perry & Ste. Genevieve Counties | 17,667 | 29 | 11 | 69 | 62 | 52 | 12 | 48 | 18 | 121 | 102 | 86 | 20 |
| 02200 | Cape Girardeau, Scott & Bollinger Counties | 21,113 | 25 | 10 | 28 | 40 | 44 | 14 | 41 | 17 | 49 | 66 | 73 | 22 |
| 02300 | Dunklin, Stoddard, New Madrid, Pemiscot & Mississippi Counties | 16,194 | 34 | 15 | 73 | 105 | 54 | 11 | 56 | 26 | 124 | 173 | 90 | 18 |
| 02400 | Butler, Ripley, Wayne, Madison, Iron, Reynolds & Carter Counties | 15,044 | 32 | 11 | 59 | 74 | 55 | 12 | 52 | 19 | 103 | 122 | 90 | 20 |
| 02500 | Howell, Texas, Wright, Douglas, Oregon, Ozark & Shannon Counties | 16,617 | 33 | 8 | 68 | 71 | 66 | 13 | 52 | 14 | 117 | 113 | 104 | 20 |
| 02601 | Christian, Greene (Outside Springfield City) & Webster Counties | 23,485 | 27 | 5 | 49 | 62 | 55 | 15 | 43 | 8 | 82 | 99 | 85 | 23 |
| 02602 | Greene County--Springfield City (North) | 24,135 | 25 | 15 | 19 | 30 | 42 | 17 | 41 | 26 | 33 | 50 | 69 | 27 |
| 02603 | Greene County--Springfield City (South) | 19,918 | 24 | 6 | 31 | 36 | 37 | 16 | 39 | 10 | 55 | 60 | 60 | 25 |
| 02700 | Taney, Barry, Stone & McDonald Counties | 20,147 | 29 | 8 | 43 | 70 | 59 | 13 | 46 | 14 | 74 | 112 | 95 | 20 |
| 02800 | Jasper & Newton Counties | 28,335 | 30 | 11 | 47 | 61 | 59 | 13 | 48 | 18 | 79 | 98 | 94 | 20 |
